# Training of Dental Professionals in Motivational Interviewing can Heighten Interdental Cleaning Self-Efficacy in Periodontal Patients

**DOI:** 10.3389/fpsyg.2016.00254

**Published:** 2016-02-24

**Authors:** Johan P. Woelber, Narin Spann-Aloge, Gilgamesh Hanna, Goetz Fabry, Katrin Frick, Rigo Brueck, Andreas Jähne, Kirstin Vach, Petra Ratka-Krüger

**Affiliations:** ^1^Department of Operative Dentistry and Periodontology, University Medical Center FreiburgFreiburg, Germany; ^2^Department of Medical Psychology, University Medical Center FreiburgFreiburg, Germany; ^3^German Academy for PsychologyBerlin, Germany; ^4^Private Psychological Practice, San DiegoCA, USA; ^5^Rhein-Jura Klinik, Clinic for PsychotherapyBad Säckingen, Germany; ^6^Department of Medical Biometry and Statistics, University Medical Center FreiburgFreiburg, Germany

**Keywords:** motivational interviewing, periodontitis, self-efficacy, oral hygiene, patient compliance

## Abstract

**Background:** The success of periodontal therapy depends on the adherence of patients to professional recommendations. The aim of this study was to investigate the influence of a workshop in motivational interviewing (MI) on non-surgical periodontal treatment performed by dental students.

**Materials and Methods:** In the experimental group patients with periodontitis were treated by students trained in MI, while in the control group patients were treated by students who had not been trained in MI. Clinical oral parameters were assessed by a blinded periodontist in addition to the evaluation of psychological questionnaires given before and after the non-surgical periodontal treatment (6 months). Conversations between patients and students were recorded and rated with the Motivational Treatment Integrity Code (MITI-d) by a blinded psychologist.

**Results:** There were 73 patients in the MI group and 99 patients in the control group. The MI group showed significantly higher scores in the MITI-d analysis. Regression analysis showed that there were no significant differences between groups with regard to plaque level, gingival bleeding, pocket depth reduction or bleeding upon probing. However, patients in the MI-group showed significantly higher interdental cleaning self-efficacy than patients in the control group (*MI* = 19.57 ± 4.7; control = 17.38 ± 6.01; *p* = 0.016).

**Conclusion:** Teaching MI to dental students resulted in a significant improvement in the self-efficacy of interdental cleaning in patients compared to a control group of non-trained students, but no improvement in other aspects of non-surgical periodontal therapy. The study also showed that an 8-h workshop with supervision significantly improved the MI-compliant conversations of dental students without requiring more conversation time.

## Introduction

The long term success of periodontal therapy is crucially dependent on the adherence of patients to therapeutic recommendations (Eickholz et al., 2008). These include adequate oral hygiene, regular follow-ups to supportive periodontal therapy, smoking cessation, control of diabetes and/or dietary recommendations (Ramseier, 2005; Eickholz et al., 2008). Therefore, periodontal therapy should include interventions to promote patient motivation. Motivational interviewing (MI), first introduced by Miller and Rollnick (2012), is a client-centerd, directive method of enhancing patients’ intrinsic motivation for behavioral change by exploring and resolving ambivalence. MI has been shown to be a suitable intervention in a clinical setting (Rollnick et al., 2008). Other studies have shown its effectiveness in modifying behavior with regard to smoking cessation, changing diets, increasing physical activity, improving body mass index and adherence to medication regimes (Wilson and Schlam, 2004; Cooperman and Arnsten, 2005; Rubak et al., 2005; Hardcastle et al., 2013; Lundahl et al., 2013). Initial studies in the field of periodontology with regard to MI showed promising but controversial outcomes (Gao et al., 2014). While studies found that MI had a positive impact on outcome for non-surgical periodontal therapy in the form of reduced pocket depth, bleeding on probing and plaque level for a period of 2 years compared to the control group (Jönsson et al., 2009, 2010), two other studies found no beneficial effects on periodontal therapy after a single session of MI (Stenman et al., 2012; Brand et al., 2013). It should be mentioned in this context that in the studies performed by Jönsson et al. (2009, 2010) MI was not the only intervention but was rather a part of a tailored oral health educational program. Furthermore, the study was performed by only a single therapist in the experimental group and a single therapist in the control group (dental hygienists) with the possible risk, that personal factors (e.g., sympathy) could have influenced the outcomes. In the studies with neutral outcomes MI was delivered by specialists in psychology with no professional background in oral hygiene. The aim of the current study was therefore to evaluate the effect of MI when administered by a cohort of dental therapists, in this case MI-trained dental students, as an adjunct to non-surgical periodontal treatment in a controlled setting over a 6-month period.

## Materials and Methods

The study was approved by the University of Freiburg Ethics Committee (EK 291/11) and registered in the German Clinical Trials Register (DRKS00003954). All patients involved in the study gave written informed consent.

### Subjects

Patients were recruited in the Department of Operative Dentistry and Periodontology of the University Freiburg Medical Center coming for periodontal treatment (initial and supportive periodontal therapy) in the student course. Patients were asked to participate by one of the authors (NSP) in order of their appearance. All participants were informed about study procedures and provided written consent upon agreement to participate. As a reward for participation the patients were treated free of charge (resulting in a mean cost savings of 70 Euros). To prevent possible intergroup influence, the control group was investigated one semester prior to the experimental group.

Both the experimental group and the control group consisted of patients treated by students taking part in the last clinical periodontal course in the curriculum (4th year). The experimental group (MI-group) started one semester after the control group.

### Inclusion Criteria

For systematic periodontal treatment, patients were required to have periodontal disease with a Community Periodontal Index for Treatment Needs (Ainamo et al., 1982) of at least two sextants with Code 3 or above.

No age criteria were applied unless patients did not fully understand the requirements of the study and the questionnaires.

### Exclusion Criteria

Patients were excluded from the study if they had any of the following characteristics: aggressive periodontitis, the presence of an infectious disease (HIV, hepatitis), pregnancy, use of antibiotics within 6 months prior to the study, xerostomia, physical inability to perform the oral hygiene procedures, or the use of drugs influencing gingival hyperplasia or bleeding.

### Procedures

After giving their written consent the patients got scheduled to an appointment with a dentist blinded to the study protocol (GH). After taking a general medical history, the dentist assessed oral hygiene indices including Plaque Index (PI, Silness and Löe, 1964) and Gingival Index (GI, Löe and Silness, 1963). Also, a full-mouth dental and periodontal examination including the measurement of pocket depth (PPD), gingival recessions, bleeding on probing (BOP), furcation involvement and mobility, was conducted. Periodontal probing was performed with a pressure-sensitive probe (DB764R, Aesculap AG, Tuttlingen, Germany). Periodontal measurements were documented with periodontal examination software (Parostatus^®^, Parostatus GmbH, Berlin, Germany) which allowed the export of the entire data set into Excel (Microsoft^®^, Redmond, USA). The dentist was trained and his practice evaluated for accuracy and reliability until the reproducibility was better than 90% (Lang et al., 2010). The examination took place in the range from 1 month to 1 day before the patient met the student.

After the clinical assessment patients then filled out questionnaires for demographic data (age, gender, level of education), oral hygiene behavior (self-rated quality of oral hygiene on a 10 point Likert scale ranging from “poor” to “very good”; four questions regarding type and frequency of oral hygiene procedures and dental visits; nine questions regarding periodontal knowledge; Woelber et al., 2015), and oral hygiene related self-efficacy assessed by a German version of the dental self-efficacy scale including self-efficacy regarding brushing, interdental cleaning and dental visits (Woelber et al., 2015). This scale included 19 items with possible values from 19 (lowest self-efficacy) to 76 (highest self-efficacy). Furthermore, the General Self-Efficacy Questionnaire (Schwarzer and Jerusalem, 1995) and the German version of the Perceived Stress Questionnaire (PSQ; Levenstein et al., 1993; Fliege et al., 2001) were administered to the patients. The patients were randomly assigned to the students by drawing lots.

**FIGURE 1:**
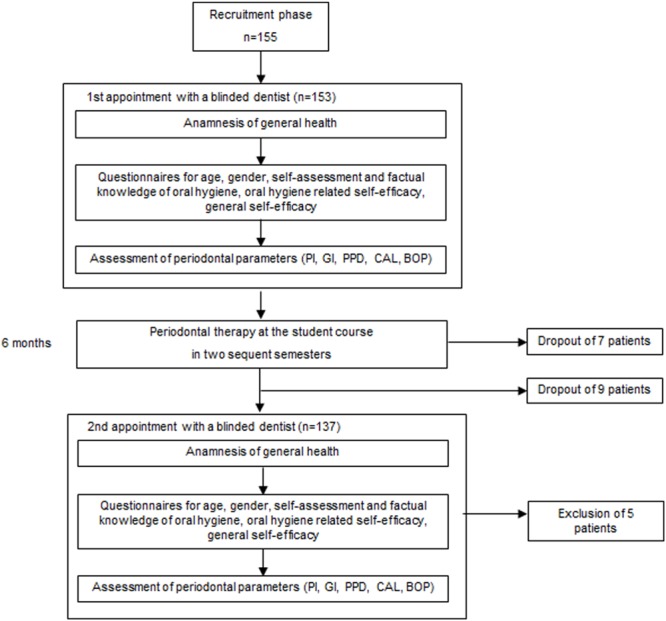
**Flow diagram of procedures**.

After completion of the student course (re-evaluation), the patient came to a second appointment for the same measurements. This took place within 1 month after the completion of the student course. **Figure 1** shows the flow diagram of procedures.

### Training in Motivational Interviewing and Quality Management

The students of the experimental group were trained in MI at an 8-h workshop by a psychologist specialized in MI (KF) at the beginning of the semester. Training included theoretical background and peer-to-peer exercises for inducing both general behavioral change as well as specific periodontal issues like improving oral hygiene or smoking cessation. Due to small group sizes limited to 20 students, the workshop was conducted twice. In addition to the training, students also received a German textbook about MI (Frick, 2010). Two weeks after the workshop students received 4-h of group supervision provided by a psychiatrist specialized in MI (AJ) in order to deepen their understanding and MI-abilities (Madson et al., 2009). They were allowed to ask questions and were invited to perform further peer-to-peer exercises with direct feedback. This training took place 1 week before their first contact with the patients.

For assessment of the student’s capabilities in performing or not-performing MI, all conversations between the students and the patients were recorded in both groups and analyzed using the German version of the MI Treatment Integrity code (MITI-d) for both the control and MI group (Moyers et al., 2005; Brueck et al., 2009). For this purpose one of the authors (JPW) was trained by a MI expert (RB) prior to the study. For quality assurance, the first fifteen conversations were rated and discussed by two raters (CS, JPW) followed by a rating of the remaining conversations by a psychologist (CS).

### Periodontal Student Course

Students of the periodontal course were asked to treat one patient coming for their initial periodontal treatment and two patients coming for supportive periodontal treatment. The non-surgical periodontal treatment (systematic treatment) consisted of 4–5 appointments with general and special anamnesis, dental, periodontal and radiographic assessment, an initial phase including oral hygiene training and professional tooth-cleaning, scaling and root planing of sites ≥4 mm, and a re-evaluation after 6–8 weeks. The duration of the appointments was approximately 2–3 h. Supportive periodontal treatment consisted of one appointment including periodontal examination, oral hygiene training and professional tooth-cleaning, scaling and root planing of sites 4 mm or deeper which showed bleeding upon probing, and were then completed with a risk assessment (Lang and Tonetti, 2003).

### Statistical Models

The null hypothesis (H_0_) was that a training in MI had no effect on clinical periodontal and oral hygiene-related parameters for short-term periodontal therapy.

Pocket depths were considered to be the primary outcome variable. Secondary outcome variables were PI, GI, clinical attachment loss, bleeding upon probing, oral hygiene related self-efficacy and oral hygiene behavior.

Sample size was limited by practicality, with semester group sizes of 33 students per semester and 3 patients per student.

The data were analyzed by a mathematician (KV) using the STATA 13.1 software (StataCorp. Ed 13. Texas, USA). For intergroup analyses the *t*-test was used. In addition, a regression analysis was performed.

## Results

Results regarding the demographic data are shown in **Table 1**. In total, 172 patients were treated by 56 students. Two patients in the control group and one patient in the MI group were excluded from analysis due to the use of antibiotics. The mean age was 59.27 years with a standard deviation of 11.40. Gender distribution showed 84 female patients (48.84%) and 88 male patients (51.16%), 39 patients smoked (22.67%), 43 patients received initial periodontal therapy (24.57%), and 132 patients supportive periodontal therapy (75.43%).

**Table 1:** Demographic characteristics of the patients.

	Control group	MI group	Total
Number of patients baseline	101	74	175
Number of patients end	99	73	172
Number of students	32	24	56
Mean age (standard deviation)	58.87 (25.99)	59.80 (10.59)	59.27 (11.40)
Gender (female/male)	48 (48.48%)/51 (51.51%)	36 (49.32%)/37 (50.68%)	84 (48.84%)/88 (51.16%)
Smoker (percentage)	23 (23.23%)	16 (21.91%)	39 (22.67%)
Initial periodontal therapy/supportive periodontal therapy	23 (23.23%)/76 (76.76%)	17 (23.28%)/56 (76.71%)	43 (24.57%)/132 (75.43%)
Mean number of teeth	22.54	23.08	22.81
Level of education (1 = Certificate of secondary education;	1 = 31 (31.31%)	1 = 22 (30.13%)	1 = 53 (30.81%)
2 = General certificate of secondary education;	2 = 22 (22.22%)	2 = 18 (24.66%)	2 = 40 (23.26%)
3 = Final secondary exam;	3 = 17 (17.17%)	3 = 11 (15.07%)	3 = 28 (16.28%)
4 = University degree;	4 = 10 (10.10%)	4 = 7 (9.59%)	4 = 17 (9.88%)
5 = Other)	5 = 19 (19.19%)	5 = 15 (20.55%)	5 = 34 (19.77%)

Oral hygiene behavior related results are shown in **Table 2**. No statistically significant difference between groups was found, with the exception of a significantly higher value for the MI-group regarding the interdental cleaning self-efficacy (*p* = 0.016). Variables were checked by qplots and showed a normal distribution.

**Table 2:** Results regarding oral hygiene, self-efficacy and stress.

	Baseline/End	Control group	MI group	*p*-value
Frequency of tooth brushing	Baseline	3.04 (0.62)	3.04 (0.57)	0.989
	End	3.09 (0.60)	3.12 (0.57)	0.779
Frequency of interdental cleaning	Baseline	2.90 (1.16)	3.13 (1.14)	0.202
	End	3.06 (1.10)	3.35 (0.83)	0.076
Frequency of dental visiting	Baseline	3.41 (0.85)	3.22 (1.14)	0.210
	End	3.53 (0.81)	3.50 (0.95)	0.850
Knowledge of oral hygiene devices	Baseline	2.13 (1.12)	2.22 (1.13)	0.615
	End	2.33 (1.09)	2.42 (1.15)	0.583
Self-rated oral hygiene	Baseline	6.66 (1.87)	7.08 (1.67)	0.155
	End	7.26 (1.20)	7.42 (1.63)	0.579
Periodontitis related knowledge	Baseline	6.63 (1.58)	6.72 (1.63)	0.726
	End	7.09 (1.54)	7.12 (1.51)	0.910
Self-efficacy regarding tooth brushing	Baseline	20.09 (5.2)	19.85 (5.24)	0.772
	End	19.70 (5.39)	21.11 (3.90)	0.078
Self-efficacy regarding interdental cleaning	Baseline	17.37 (6.20)	16.67 (6.06)	0.475
	End	17.38 (6.01)	19.57 (4.70)	0.016
Self-efficacy regarding dental visiting	Baseline	22.27 (6.50)	21.42 (7.13)	0.436
	End	22.96 (6.27)	23.49 (6.29)	0.610
General self-efficacy	Baseline	31.92 (5.30)	31.85 (3.67)	0.929
	End	31.46 (4.84)	31.06 (4.10)	0.586
Stress	Baseline	31.98 (7.00)	30.05 (14.36)	0.447
	End	29.93 (16.50)	31.60 (19.40)	0.561

The clinical results are shown in **Table 3**. In total, BOP, clinical attachment level (CAL) and PPD improved in both groups. Plaque values increased slightly in both groups (MI group: 0.18 ± 0.28; control group: 0.09 ± 0.31; *p* = 0.091), while the gingival index dropped in the experimental group (-0.06 ± 0.29) and increased in the control group (0.14 ± 0.27). Analysis showed significantly higher reduction of GI values in the MI group compared to the control group (*p* < 0.001). Furthermore, the MI-group showed significantly higher reduction of pocket probing depths on average compared to the control group (MI group: -0.75 ± 0.64; control group: -0.54 ± 0.60; *p* = 0.035).

**Table 3:** Clinical results baseline and after the non-surgical periodontal therapy.

	Baseline/End	Control group	MI group	*p*-value
GI	Baseline	0.91 (0.27)	1.10 (0.15)	
	End	1.05 (0.15)	1.03 (0.29)	
	Difference	+0.14 (0.27)	-0.06 (0.29)	0.000
PI	Baseline	0.43 (0.30)	0.56 (0.30)	
	End	0.54 (0.32)	0.72 (0.32)	
	Difference	+0.09 (0.31)	+0.18 (0.28)	0.091
BOP	Baseline	53.65 (23.86)	51.87 (23.18)	
	End	51.82 (27.32)	46.65 (25.07)	
	Difference	-1.84 (25.04)	-5.23 (25.88)	0.402
CAL	Baseline	5.23 (0.96)	3.42 (2.53)	
	End	4.81 (1.19)	3.17 (2.36)	
	Difference	-0.42 (0.77)	-0.25 (0.56)	0.112
PPD > 6 mm in percent	Baseline	3.40% (5.67)	10.12% (18.93)	
	End	4.94% (8.46)	7.32% (11.02)	
	Difference	+1.54% (7.06)	-2.80% (14.98)	0.072
PPD 4–6 mm in percent	Baseline	95.59% (11.23)	89.88% (18.93)	
	End	46.69% (23.81)	47.81% (19.46)	
	Difference	-48.90% (24.80)	-41.46% (28.71)	0.305
PPD mean (≥4 mm)	Baseline	4.45 (0.34)	4.66 (0.59)	
	End	3.91 (0.69)	3.90 (0.73)	
	Difference	-0.54 (0.60)	-0.75 (0.64)	0.035

*Results are presented in mean and standard deviation in parenthesis. GI, gingiva index; PI, plaque index; BOP, bleeding on probing; CAL, clinical attachment loss; PPD, pocket probing depth.*

Due to the differences in baseline values between groups a regression analysis was performed, whose results are presented in **Table 4**. Regression analysis revealed that the changes in interdental cleaning self-efficacy were significantly related to group affiliation (MI vs. control group; *p* = 0.017), and that the changes in GI were significantly related to the patient’s gender (*p* = 0.025), as well as whether the patient was coming for systematic or supportive periodontal treatment (*p* < 0.001). This latter factor also had a significant impact on the CAL (*p* = 0.042). The changes in the plaque index were significantly associated to group affiliation (MI- vs. control group; *p* = 0.003) and smoking (*p* = 0.009).

**Table 4:** Regression analysis with *p*-values regarding different variable changes.

Variable	Group (control/MI)	Smoking	Age	Gender	Initial or supportive periodontal therapy
Self-efficacy regarding interdental cleaning	0.017	0.715	0.582	0.089	0.163
GI	0.215	0.444	0.367	0.025	0.000
PI	0.003	0.009	0.459	0.054	0.243
BOP	0.301	0.320	0.504	0.436	0.169
CAL	0.752	0.979	0.321	0.649	0.042
PPD	0.081	0.620	0.334	0.711	0.587

*GI, gingival index; PI, plaque index; BOP, bleeding on probing; CAL, clinical attachment loss; PPD, pocket probing depth.*

Results regarding the MITI-d are shown in **Table 5**. Almost all factors were significantly higher in the experimental group except the time of behavior-related conversation (up to 20 min; *p* = 0.311), the amount of information giving by the student (*p* = 0.235), and the number of complex reflections (*p* = 0.036).

**Table 5:** MITI-d analysis of the recorded conversations.

	Control group	MI group	*p*-value
Time of behavior-related conversation (up to 20 min) [min]	11.07 (5.72)	11.98 (6.23)	0.311
Empathy	1.55 (1.03)	2.91 (2.27)	<0.001
MI spirit	2.00 (1.16)	3.78 (2.33)	<0.001
MI adherent communication	3.61 (2.63)	5.02 (3.80)	<0.001
MI non-adherent communication	2.81 (3.30)	1.55 (1.83)	0.033
Giving information	10.10 (5.49)	10.64 (6.36)	0.235
Closed questions	4.90 (3.43)	6.05 (5.18)	0.008
Open questions	1.13 (1.28)	2.62 (2.82)	<0.001
Simple reflections	0.38 (0.72)	0.85 (1.25)	0.009
Complex reflections	0.05 (0.29)	0.36 (1.00)	0.069
Total reflections	0.43 (0.75)	1.21 (1.96)	0.007
Open questions %	17.57 (19.05)	28.91 (24.53)	0.005
Complex reflections %	3.33 (18.10)	10.46 (23.76)	0.036
MI adherent %	61.00 (29.38)	73.57 (30.42)	0.024
Ratio of open questions to closed questions %	0.31 (0.51)1:3	0.49 (0.54)1:2	0.031
Ratio of reflections to questions %	0.08 (0.27)	0.12 (0.18)	0.021

*MI, Motivational Interviewing.*

## Discussion

The present study aimed to evaluate the effect of a workshop in MI for dental therapists on patients coming for non-surgical periodontal therapy over a 6 months period. In order to be able to assess a large number of therapists in a controlled setting the study was performed in a periodontal student course. Statistical analysis showed significant differences between the groups for parameters such as the gingival index and mean reduction in pocket probing depth in favor of the MI group. Due to a difference in baseline clinical values a further regression analysis was performed. This analysis showed that the final results were not caused by group affiliation, except for the changes in oral plaque values and oral hygiene-related self-efficacy. In this context, the MI group showed a significantly greater increase in the self-efficacy of interdental cleaning. This finding is interesting because this factor was shown to be highly correlated with current and prospective oral hygiene behavior (Syrjälä et al., 1999; Kakudate et al., 2010; Lee et al., 2012; Woelber et al., 2015). It can be assumed that the main focus of students was to influence oral hygiene behavior in their patients. If MI was effective in improving the self-efficacy of oral hygiene, it may also be an effective instrument in improving the self-efficacy of other periodontal risk factors such as smoking, nutrition or diabetic control (Macnee and Talsma, 1995; Skelly et al., 1995; Shannon et al., 1997; Fletcher and Banasik, 2001).

Looking more closely at the regression analysis of the changes in pocket depth, clinical attachment level and bleeding upon probing, none of the analyzed variables (group affiliation, gender, age, initial periodontal treatment or supportive periodontal therapy) had a significant effect. These results are consistent with other findings (Stenman et al., 2012) which did not report an effect for a single MI session performed by an MI therapist on clinical periodontal parameters over a 6 months period. It can be hypothesized that scaling and root planing had a greater influence on these parameters over the 6 months timeframe of the study than factors that had no direct effect on the subgingival biofilm. This assumption is supported by findings demonstrating the effectiveness of scaling and root planing alone in comparison to additional treatment options (Goodson et al., 2012). According to this pronounced short-term effect of scaling and root planing, the duration of the study was probably too short to detect potential clinical effects of MI, and extension of the study duration should be considered in future studies. Due to the infrastructure of the university periodontal curriculum, it was not possible to prolong the student-patient contact. Longer therapist-patient contact and 1-year study duration could be important factors in the positive results of those studies showing favorable MI effects in periodontology (Jönsson et al., 2009, 2010). Furthermore, patient compliance with periodontal follow-up can only be assessed in long-term studies. In a study with a 10-year follow-up of periodontal patients, Eickholz et al. (2008) found that the most important risk factor for tooth loss was lack of patient compliance with supportive periodontal therapy.

Some comments should be made on oral hygiene parameters. Both groups showed a slight increase of plaque values at the end of the study, but there was no significant difference between the groups. This may be due to the comparatively long interval between completion of the student course and the dental re-evaluation, which might have led to a relapse in oral hygiene behavior (Tedesco et al., 1992). Regression analysis showed that there was a significant association between the increase of plaque values and MI group affiliation. The MI group also showed a significant improvement in gingival bleeding compared to the control group. It can be hypothesized that the higher plaque values in the MI group were due to the higher proportion of deep pockets (>6 mm) than in the control group, causing more inflammation and more plaque (Rowshani et al., 2004). Furthermore, it also needs to be discussed whether marginal bleeding may be a more important factor than plaque, due to its long-term effects (Löe et al., 1965).

Another important aspect is the quality of the MI performed by the students. The results show clear effects for a 1-day MI workshop with additional educational literature and a supervisory session. The MITI-d analysis revealed that all important MI variables were significantly higher in the MI-trained students’ conversations compared to those of the untrained students. However, MI-trained students did not reach the base level normally recommended for MI therapists (**Table 6**), although it should be noted that these base levels are based on an expert opinion (Moyers et al., 2005). It can be assumed that the clinical and psychological effects that were detected would have been much more pronounced if the students had achieved the level recommended for MI-therapists. This issue should be addressed in future studies. The duration of training (8 h) was similar to that of other studies in the field of medical health care (Madson et al., 2009). A study involving a more intensive, 2-day MI training session for clinicians in the field of substance abuse showed comparable and slightly better results in the MITI analysis (Smith et al., 2007). It cannot be expected that one or two workshops will create an MI expert. As Miller and Moyers (2006) stated, learning MI is not a simple but rather a continuous process, with different stages of training in philosophy, attitudes and practical skills. It is also important to consider the extent to which MI is applicable in a traditional dental setting, which involves many directive instructions (e.g., ‘open your mouth’ or ‘rinse out’). Looking at the length of the conversations, there was no difference in the duration of behavior-related communication between groups. This shows that even if patients in the MI group speak for a longer amount of time (due to more open questions) than those in control group, it does not necessarily mean that the total time of conversation is longer.

**Table 6:** Comparison between the groups regarding MITI-d analysis and recommended basic values for MI-therapists (Moyers et al., 2005).

	Control group	MI group	Recommendations for MI therapists
Global values (empathy/MI spirit)	1.76	3.35	5
MI adherent communication in Percent	61%	74%	90%
Open questions in percent	18%	29%	50%
Complex reflections in percent	3%	10%	40%
Ratio of reflections to questions	0.08:1	0.12:1	1:1

Regarding the practical implications of implementing MI in a dental curriculum, further studies are needed regarding both the clinical effects of MI and the efficiency of educational methods. A study by Schoonheim-Klein et al. (2013) compared three different kinds of MI training for dental students and found the most pronounced effects with a 4-h workshop and an additional 60 min of role-playing, followed by an objective structured clinical examination (OSCE). This shows that the effects of teaching MI can be enhanced by examinations. Another study by DeBate et al. (2012) found positive effects of a brief MI e-learning tool on the skill-based knowledge of students. Although no conversation skills were measured by means of MITI, e-learning seems to be a promising step for teaching the knowledge-based aspects of MI.

The original study plan included a second control group with sham exposure for the students, but this had to be omitted due to limited funding. Sham exposure would have involved a training session in communication without MI-specific elements. Gao et al. (2014) were unable to identify any studies about MI in a dental setting that included a sham exposure, so this would be a worthwhile feature of further studies. Furthermore, patient assessments of student empathy were discontinued after the first 20 questionnaires since they consistently awarded the highest possible score. It can be assumed that the patients wanted to protect their personal students from negative consequences. The main limitation of the aspect of the study design relating to clinical outcomes was that the experimental group was assessed after the control group. This allows possible confounders such as different student abilities or differences in patient behavior across the seasons (e.g., autumn vs. spring). However, to the best of our knowledge this is the first study to incorporate such a high number of MI-trained and non-MI-trained therapists within a dental setting.

## Conclusion

Within the limitations, this study showed that teaching MI to dental students resulted in a significant improvement in the self-efficacy of interdental cleaning for patients as compared to a control group of non-trained students, but no improvement in other aspects of non-surgical periodontal therapy. The study also showed that an 8-h workshop including supervision significantly improved the MI-compliant conversations of dental students without requiring more conversation time.

## Author Contributions

JPW contributed to planning and conduction of the study and writing the manuscript. NSP contributed to planning and conduction of the study and writing the manuscript. GH contributed to conduction of the study and writing the manuscript. GF contributed to planning of the study and writing the manuscript. KF contributed to planning and conduction of the study and writing the manuscript. RB contributed to planning of the study and writing the manuscript. AJ contributed to conduction of the study and writing the manuscript. KV contributed to planning and data analysis of the study and writing the manuscript. PRK contributed to planning and conduction of the study and writing the manuscript.

## Conflict of Interest Statement

The authors declare that the research was conducted in the absence of any commercial or financial relationships that could be construed as a potential conflict of interest.
